# Supplementary material for: A role for glial fibrillary acidic protein (GFAP)-expressing cells in the regulation of gonadotropin-releasing hormone (GnRH) but not arcuate kisspeptin neuron output in male mice
Source: eLife. 2021 Jul 22;10:e68205. doi: 10.7554/eLife.68205 (PMC8337074; doi:10.7554/eLife.68205)
Supplement: Transparent reporting form [file elife-68205-transrepform.pdf]

1st Floor
24 Hills Road
Cambridge CB2 1JP, UK

P 01223 855340
W elifesciences.org
T @elife

eLife's transparent reporting form

We encourage authors to provide detailed information *within their submission* to facilitate the interpretation and replication of experiments. Authors can upload supporting documentation to indicate the use of appropriate reporting guidelines for health-related research (see EQUATOR Network), life science research (see the BioSharing Information Resource), or the ARRIVE guidelines for reporting work involving animal research. Where applicable, authors should refer to any relevant reporting standards documents in this form.

If you have any questions, please consult our Journal Policies and/or contact us: editorial@elifesciences.org.

Sample-size estimation

- You should state whether an appropriate sample size was computed when the study was being designed
- You should state the statistical method of sample size computation and any required assumptions
- If no explicit power analysis was used, you should describe how you decided what sample (replicate) size (number) to use

Please outline where this information can be found within the submission (e.g., sections or figure legends), or explain why this information doesn't apply to your submission:

For electrophysiology, numbers are based on power analysis in which historic mean differences and standard deviations were used for a variety of data types (e.g., firing rate, synaptic transmission). A sample size of 8-15 cells/group depending on parameter has an 85% power to detect a difference between means of 30% (minimum considered biologically relevant) with a significance level (alpha) of 0.05 (two-tailed) in an unpaired t-test. From these calculations we established a range of 8-15 quality recordings per group. For the validation of animal models, the sample size was smaller (n=3 to 6).

Replicates

- You should report how often each experiment was performed
- You should include a definition of biological versus technical replication
- The data obtained should be provided and sufficient information should be provided to indicate the number of independent biological and/or technical replicates
- If you encountered any outliers, you should describe how these were handled
- Criteria for exclusion/inclusion of data should be clearly stated
- High-throughput sequence data should be uploaded before submission, with a private link for reviewers provided (these are available from both GEO and ArrayExpress)

Please outline where this information can be found within the submission (e.g., sections or figure legends), or explain why this information doesn't apply to your submission:

The number of biological and technical replicates is reported in the text for each experiment. It is reported in the materials and methods, in the results and in table 3.

Statistical reporting

- Statistical analysis methods should be described and justified
- Raw data should be presented in figures whenever informative to do so (typically when N per group is less than 10)
- For each experiment, you should identify the statistical tests used, exact values of N, definitions of center, methods of multiple test correction, and dispersion and precision measures (e.g., mean, median, SD, SEM, confidence intervals; and, for the major substantive results, a measure of effect size (e.g., Pearson's r, Cohen's d)
- Report exact p-values wherever possible alongside the summary statistics and 95% confidence intervals. These should be reported for all key questions and not only when the p-value is less than 0.05.

Please outline where this information can be found within the submission (e.g., sections or figure legends), or explain why this information doesn't apply to your submission:

The description of statistical analysis methods used is reported in the 'Analysis and Statistics' part of the materials and methods section. Mean and SEM for each data sets are reported in the results and in the figures. The statistical tests and parameters used together with P values are reported in the results as well as in tables 4, 5 and 6.

(For large datasets, or papers with a very large number of statistical tests, you may upload a single table file with tests, Ns, etc., with reference to sections in the manuscript.)

Group allocation

- Indicate how samples were allocated into experimental groups (in the case of clinical studies, please specify allocation to treatment method); if randomization was used, please also state if restricted randomization was applied
- Indicate if masking was used during group allocation, data collection and/or data analysis

Please outline where this information can be found within the submission (e.g., sections or figure legends), or explain why this information doesn't apply to your submission:

Experiments were performed blinded whenever possible. Animals were distributed randomly among groups. This information is reported in the materials and methods section of the paper.

Additional data files ("source data")

- We encourage you to upload relevant additional data files, such as numerical data that are represented as a graph in a figure, or as a summary table
- Where provided, these should be in the most useful format, and they can be uploaded as "Source data" files linked to a main figure or table
- Include model definition files including the full list of parameters used
- Include code used for data analysis (e.g., R, MatLab)
- Avoid stating that data files are "available upon request"

Please indicate the figures or tables for which source data files have been provided:

Individual data points are presented in the figures for every *in vivo* measurement and every electrical recording. Analysis software for calcium signals and event detection have been deposited to GitLab and are accessible at https://gitlab.com/um-mip/coding-project
